# Relationship between menopause and health-related quality of life in middle-aged Chinese women: a cross-sectional study

**DOI:** 10.1186/1472-6874-14-7

**Published:** 2014-01-10

**Authors:** Kuo Liu, Liu He, Xun Tang, Jinwei Wang, Na Li, Yiqun Wu, Roger Marshall, Jingrong Li, Zongxin Zhang, Jianjiang Liu, Haitao Xu, Liping Yu, Yonghua Hu

**Affiliations:** 1 Department of Epidemiology and Biostatistics, Peking University Health Science Center, Beijing, China; 2 Chinese Center for Disease Control and Prevention, Beijing, China; 3 Fangshan District Bureau of Health, Beijing, China; 4 Department of Statistics, The University of Auckland, Auckland, New Zealand; 5 The First Hospital of Fangshan District, Beijing, China

## Abstract

**Background:**

Chinese menopausal women comprise a large population and the women in it experience menopausal symptoms in many different ways. Their health related quality of life (HRQOL) is not particularly well studied. Our study intends to evaluate the influence of menopause on HRQOL and explore other risk factors for HRQOL in rural China.

**Methods:**

An interview study was conducted from June to August 2010 in Beijing based on cross-sectional design. 1,351 women aged 40–59 were included in the study. HRQOL was measured using the EuroQol Group’s 5-domain (EQ5D) questionnaire. Comparison of HRQOL measures (EQ5D index and EQ5D-VAS scores) was done between different menopausal groups. Logistic regression and multiple regression analysis were performed to adjust potential confounders and explore other risk factors for health problems and HRQOL measures.

**Results:**

Postmenopausal women who had menopause for 2–5 years (+1b stage) were more likely to suffer mobility problems (OR = 1.835, *p* = 0.008) after multiple adjustment. Menopause was also related to impaired EQ5D index and EQ5D-VAS scores after adjustment for age. Among menopausal groups categorized by menopausal duration, a consistent decrement in EQ5D index and EQ5D-VAS scores, that is, worsening HRQOL, was observed (*p* < 0.05). Multiple regression analysis revealed low education level and physical activity were associated with EQ5D index (β = -0.080, *p* = 0.003, and β = 0.056, *p* = 0.040, respectively). Cigarette smoking and chronic disease were associated with EQ5D index (β = -0.135, *p* < 0.001 and β = -0.104, *p* < 0.001, respectively) and EQ5D-VAS (β = -0.057, *P* = 0.034 and β = -0.214, *p* < 0.001, respectively).

**Conclusions:**

Reduction in physical function was found within the first five years after menopause. Worsening EQ5D index and EQ5D-VAS scores were related to menopause. Education level, physical activity, cigarette smoking, and chronic disease history were associated with HRQOL in middle aged Chinese rural women.

## Background

Health-related quality of life (HRQOL) is an essential feature of health care. It represents many factors that affect individual’s health, including physical, psychological, social aspects and subjective experience. The EuroQol Group’s 5-domain questionnaire, EQ-5D, is a generic measure of HRQOL [1]. The EQ-5D has been translated into several languages, including Chinese, and its validity has also been determined [2]. These advantages make the EQ-5D widely used in different countries to access health-state preference in general population and specific patient groups, including menopausal women [3-5].

Menopause is a normal degenerative transition associated with aging and loss of fertility [6]. Women during menopause experience not only biological changes but also social and cultural changes [7]. These changes make them more vulnerable to physical health problems and mental health disorders [8,9]. Several studies in western countries have demonstrated that menopause related symptoms may impact health [10,11]. However, physical and mental symptoms during menopause may be different in Chinese. Chinese women report significantly lower psychosomatic and vasomotor symptoms during menopause than Caucasian and African-American [12,13], and Chinese menopausal women have half the risk of depressive symptoms as white women [14]. The differences of menopausal symptoms suggest that the impact of menopause on HRQOL may also be different in China. Although western studies have shown women experiencing menopause may have impaired HRQOL [11,15-17], we cannot necessarily draw the same conclusion in a Chinese population. In a study from Taiwan, Kinmen found that peri- and postmenopausal women had lower HRQOL compared to postmenopausal women based on their baseline data [18]. However, after 2 years follow up, no significant effect was found of menopausal transition on quality of life among Taiwanese women [19].

In mainland China, the overall median age at menopause is 50 years [20]. The number of rural women older than 50 was about 78 million in 2000 [21]. This number will increase to nearly 100 million in 2030 [22]. This large population, and different social environment from Taiwan, merits a further study of mainland Chinese women. In the present study, we examine whether menopause has a negative impact on HRQOL among middle-aged women and explore other characteristics which may have impact on HRQOL in rural China.

## Methods

### Study population and data sources

This cross-sectional survey was conducted from June to August 2010 in Fangshan, China. The inclusion criteria for target population are native permanent residents aged over 40 and lived in local communities for at least 5 years. A stratified clustered sampling method was employed. 1351 women aged 40–59 who was naturally menopause were evaluated in the present study. This study was approved by the ethics Committee of Peking University Health Sciences Center. Each participant signed an informed consent prior to be part of the study.

### Measures

A questionnaire was used to collect patient information during a face-to-face interview in the participant’s residential area. The questionnaire included socio-demographic characteristics (age, marital status, and educational background), health-related variables (chronic diseases history), reproductive factors (menopausal status and menopausal age) and lifestyle factors (physical activity, cigarette smoking and alcohol consumption).

Women were asked whether their menstruation had stopped naturally for at least one year without hormone therapy. Women without menopause for at least one year were asked whether their menstrual period was regular. Women having regular menstrual cycles in the previous year and menstruation within the previous 33 days were defined as premenopausal. Postmenopausal was defined as at least 12 months of amenorrhea without bilateral oophorectomy, simple hysterectomy, hormone therapy or pregnancy. The speed of hormone changes and bone loss varies in the early and the late postmenopause [23]. Postmenopausal women were divided into two groups according to the Stages of Reproductive Aging Workshop (STRAW) classification in order to identify the differences in HRQOL with different menopausal stages [23]. Postmenopausal women, menopause ≥ 1 year but within the first five years after menopause, were in the early stage of menopause (+1b stage). Women who had menopause for more than five years were in the late stages of menopause (+2 stage).

Health-related quality of life (HRQOL) was assessed using the EuroQol EQ-5D and visual analog scale of EQ5D (EQ-VAS). Lower values of these indices indicate worse HRQOL. The EQ5D questionnaire contains 3 levels evaluating mobility, self-care, usual activity, pain and anxiety/depression. The EQ5D index based score was generated by applying societal preference weights to each of the above five health dimensions according to a Japan population-based time trade-off (TTO) model [24]. In addition, a 0–100 visual scale (100, best imaginable health state; 0, worst imaginable state of health) was used to assess self-reported current health status.

Demographic characteristics (age, sex, education, and marriage status) and lifestyle risk factors (smoking status, alcohol drinking and physical activity) were also collected. Education was categorized by whether high school was attended or not. People not married included those who were single, divorced or widowed. Smoking status was measured through three alternatives: “currently smoking”, “past smoking”, or “rare/never smoking”. Current smoking was defined as having smoked more than 100 cigarettes and still smoking during the last month. Past smoking was defined as having smoked for at least one month. Rare/never smoking was defined as having smoked less than 100 cigarettes lifetime. Drinking status was measured using three alternatives: “currently drinking”, “past drinking”, and “rare/never drinking”. People who drank at least once per week and still drank at that frequency in the previous month were defined as current drinkers. Those who drank previously, but did not drink in the previous month, were defined as past drinkers. Rare/never drinking was defined as never drinking more than once a week. Physical activity was defined as light to intense exercise of at least 30 minutes per time. The frequency of physical activity was measured as: rarely/never, 1–4 times/week, or ≥ 5 times/week.

Chronic disease status was defined as having been diagnosed at least one of the following diseases: hypertension, coronary heart disease (CHD), type 2 diabetes mellitus (T2DM), stroke, or having been treated regularly for those diseases. Hypertension was defined as systolic blood pressure (SBP) ≥140 mmHg, or diastolic blood pressure (DBP) ≥90 mmHg, or current use of any antihypertensive medication or any combination of the above situations. CHD was defined as an angina or hospitalization history for myocardial infarction with electrocardiography (ECG) findings, or a surgical history of coronary balloon angioplasty, coronary artery bypass or coronary stent implantation. In addition, people having been diagnosed as T2DM in hospitals, or self-reported current treatment with insulin or oral hypoglycemic drugs were defined as T2DM. Stroke was defined as a history of language or physical dysfunction which had been continued for more than 24 h and diagnosed using computerized tomography (CT) or magnetic resonance imaging (MRI).

### Statistical methods

Analysis of various (ANOVA) and Chi-square tests was used to compare continuous and qualitative data among the three different menopause groups. Health quality, which was measured by EQ5D-VAS scores or EQ5D index, was adjusted for potential confounders by multiple linear regressions before doing comparisons between premenopausal and postmenopausal group. T-tests were used to compare difference of EQ-5D index and EQ-VAS scores between premenopausal and postmenopausal group. Health problems were classified into two categories based on the severity of problem (0 = no problem, 1 = mild to severe problem). The “problems” considered were: mobility, health problems in self-care, pain, and anxiety/depression. Logistic regression and multiple linear regressions were used to explore risk factors (including menopausal stages) for health problems and health quality, which were the outcome variables in the regression. Odds ratios (OR), 95% confidence intervals (CI) and *p* value were obtained from logistic regression analysis. Regression coefficients, standard errors and *p* value were obtained from multiple regression analysis. Since health problems and quality of life may change with age, and since menopause onset is also age related, all regressions were adjusted for age, treating age as a continuous variable. Analyses were performed using SPSS (version 18.0) software. All tests were two-sided and <0.05 was considered statistically significant.

## Results

### General characteristics of subjects

There were 1,812 eligible women, of whom 1,419 agreed to take part, a response rate of 78.3%. All 1,419 women aged 40 ~ 59 were investigated from June to August 2010 in Fangshan. After excluding 68 women whose menstruation was not naturally stopped, 1,351 women aged 40–59 that met the entry criteria were evaluated, 656 women were premenopausal, 133 women had menopause over two menstrual cycles but less than one year and 562 women were postmenopausal. Among these postmenopausal women, 276 women had menopause for more than 2 years but less than 5 years, and 286 women had menopause for more than 5 years.

The average age at menopause was 49.4 ± 3.7 years among postmenopausal women and their menopause duration was 5.1 ± 3.9 years. 82.8% of women in our study did not have high school education. Most women (over 90%) never smoked and only 4.0% of women drank alcohol. 32.7% of women exercised over five times per week and 54.7% of women never or rarely did physical exercise. 1.6% of women had a history of stroke, 4.5% coronary heart disease, 8.7% diabetes, and 30.7% hypertension. Demographic characteristics, life style and chronic disease history were similar among each group categorized by menopausal status, except for age (Table 1).

**Table 1:** Demographic characteristics, health behavior and chronic disease history among middle aged Chinese women

	**Premenopausal**	**Menopause 0 ~ 1 year**	**Postmenopausal**	***P *** **value**
Age	46.502 ± 3.503	51.421 ± 3.572	55.15 ± 2.961	<0.001^***^
Education				0.074
≤high school	542 (82.7 %)	119 (89.5 %)	452 (81.1 %)	
>high school	113 (17.3 %)	14 (10.5 %)	105 (18.9 %)	
Marriage				0.644
Yes	623 (95.1 %)	126 (94.7 %)	523 (93.9 %)	
No	32 (4.9 %)	7 (5.3 %)	34 (6.1 %)	
Smoking Status				0.510
Never	605 (92.2 %)	122 (91.7 %)	502 (90.1 %)	
Current	46 (7.0 %)	11 (8.3 %)	52 (9.3 %)	
Past	5 (0.8 %)	0 (0.0 %)	3 (0.6 %)	
Alcohol Drinking				0.553
Never	624 (95.3 %)	129 (97.0 %)	537 (96.4 %)	
Current	29 (4.4 %)	4 (3.0 %)	20 (3.6 %)	
Past	2 (0.3 %)	0 (0.0 %)	0 (0.0 %)	
Physical activities				0.058
Rarely/never	349 (53.2 %)	89 (66.9 %)	298 (53.6 %)	
1 ~ 4 times/week	83 (12.7 %)	13 (9.8 %)	74 (13.3 %)	
≥5 times/week	224 (34.1 %)	31 (23.3 %)	184 (33.1 %)	
Stroke				0.353
No	649 (98.9 %)	130 (97.7 %)	546 (98.0 %)	
Yes	7 (1.1 %)	3 (2.3 %)	11 (2.0 %)	
Hypertension				0.402
No	458 (69.8 %)	98 (73.7 %)	378 (67.9 %)	
Yes	198 (30.2 %)	35 (26.3 %)	179 (32.1 %)	
CHD				0.103
No	624 (95.1 %)	123 (92.5 %)	538 (96.6 %)	
Yes	32 (4.9 %)	10 (7.5 %)	19 (3.4 %)	
Diabetes				0.559
No	593 (90.4 %)	123 (92.5 %)	512 (91.9 %)	
Yes	63 (9.6 %)	10 (7.5 %)	45 (8.1 %)	

***p < 0.001.

### Menopause and other factors associated with health problems

Logistic regression was used to find whether postmenopausal women had more health problems after adjustments for only age or age together with chronic disease status (Table 2). Compared to premenopausal women, postmenopausal women reported mobility problems after adjusting only for age, or age and chronic diseases (OR > 2, *p* < 0.05). Health problems in self-care, usual activity, pain, and anxiety/depression dimensions were not statistically different between premenopausal and postmenopausal women regardless of potential confounders.

**Table 2:** **Different adjustment of odds ratios (95**%**CI) of menopause with health problems**

	**Crude OR**	**Age adjusted OR**	**Age and chronic diseases status adjusted OR**
Mobility	1.597 (1.108, 2.301)^*^	2.220 (1.201, 4.102)^*^	2.301 (1.241, 4.266)^**^
Self-care	1.280 (0.726, 2.255)	1.370 (0.532, 3.530)	1.439 (0.557, 3.717)
Usual activities	1.304 (0.893, 1.904)	1.700 (0.901, 3.207)	1.784 (0.941, 3.383)
Pain	1.221 (0.941, 1.584)	1.501 (0.971, 2.319)	1.520 (0.983, 2.350)
Anxiety/depression	0.836 (0.566, 1.236)	1.164 (0.611, 2.217)	1.167 (0.613, 2.224)

*p < 0.05, **p < 0.01.

In order to find in which menopausal stages women had more health problems, and explore other independent risk factors of health problems, logistic regressions were performed using age, menopause stages, marriage, education level, cigarette smoking, physical activity, and chronic disease status as independent variables (Table 3). Women who had menopause for 2–5 years reported more mobility problems (OR = 1.835, *p* = 0.008). Menopause for less than one year was associated with self-care (OR = 2.371, *p* = 0.025) and usual activity (OR = 1.867, *p* = 0.007) problems. Smoking was related to mobility problems (OR = 2.30, *p* < 0.001), self-care (OR = 2.28, *p* < 0.001) problems, usual activity problems (OR = 1.87, *p* = 0.007) and anxiety/depression problems (OR = 1.84, *p* = 0.012). Women without high school education experienced more mobility problems and usual activity problems (OR = 1.92, *p* = 0.027; OR = 2.64, *p* = 0.004, respectively) and more pain problems (OR = 1.48, *p* = 0.031) than those with high school education.

**Table 3:** Logistic regression analysis for each health dimension in EQ5D

		**OR**	**95%CI**	***P *** **value**
Mobility	Education	1.924	1.078, 3.433	0.027^*^
	Chronic disease	1.904	1.332, 2.722	<0.001^***^
	Cigarette smoking	2.298	1.495, 3.530	<0.001^***^
	Premenopausal	1.000	-	-
	Menopause			
	0 ~ 1 years	1.529	0.849, 2.755	0.157
	2 ~ 5 years	1.835	1.172, 2.873	0.008^**^
	>5 years	1.373	0.862, 2.186	0.181
Self-care	Chronic disease	2.920	1.654, 4.794	<0.001^***^
	Cigarette smoking	2.281	1.262, 4.124	0.006^**^
	Physical activity	0.856	0.735, 0.997	0.046^*^
	Premenopausal	1.000	-	-
	Menopause			
	0 ~ 1 years	2.371	1.117, 5.032	0.025^*^
	2 ~ 5 years	1.023	0.478, 2.185	0.954
	>5 years	1.389	0.710, 2.718	0.337
Usual activities	Education	2.640	1.356, 5.139	0.004^**^
	Chronic disease	2.324	1.618, 3.337	<0.001^***^
	Cigarette smoking	1.867	1.184, 2.945	0.007^**^
	Premenopausal	1.000	-	-
	Menopause			
	0 ~ 1 years	1.855	1.061, 3.242	0.030^*^
	2 ~ 5 years	1.092	0.662, 1.803	0.729
	>5 years	1.468	0.934, 2.307	0.096
Pain	Education	1.481	1.037, 2.117	0.031^*^
	Premenopausal	1.000	-	-
	Menopause			
	0 ~ 1 years	1.371	0.903, 2.082	0.139
	2 ~ 5 years	1.219	0.877, 1.694	0.238
	>5 years	1.235	0.893, 1.707	0.201
Anxiety/depression	Cigarette smoking	1.843	1.143, 2.971	0.012^*^
	Physical activity	0.894	0.804, 0.995	0.041^*^
	Premenopausal	1.000	-	-
	Menopause			
	0 ~ 1 years	0.895	0.465, 1.723	0.895
	2 ~ 5 years	0.767	0.452, 1.302	0.327
	>5 years	1.040	0.647, 1.627	0.871

*p < 0.05, **p < 0.01, ***p < 0.001.

### Menopause and other factors contributing to health quality

Health quality, which was measured by EQ5D index and EQ5D-VAS, were firstly compared between premenopausal and postmenopausal group. After adjusting only for age, age and chronic diseases, t-tests showed similar results (Table 4): postmenopausal women had lower EQ5D index and EQ5D-VAS scores than premenopausal women (*p* < 0.001).

**Table 4:** Means and standard deviation of EQ-5D and EQ-VAS categorized by menopause status after different adjustments

	**Unadjusted**	**Age adjusted**	**Age and chronic diseases status adjusted**
**EQ5D index**			
premenopausal group	0.810 (0.070)	0.806 (0.004)	0.806 (0.009)
postmenopausal group	0.800 (0.085)	0.802 (0.003)	0.804 (0.009)
*P* value	0.005^**^	<0.001^***^	<0.001^***^
**EQ5D-VAS**			
premenopausal group	71.819 (17.053)	72.387(0.240)	72.157 (3.638)
postmenopausal group	72.142 (17.112)	71.801 (0.205)	71.746 (3.626)
*P* value	0.742	<0.001^***^	0.049^*^

* p < 0.05, **p < 0.01, ***p < 0.001.

A stepwise multivariate linear regression analysis was performed to find which stages of menopause had lower health quality, and explore other risk factors for health quality using age, marriage, education level, cigarette smoking, physical activity, and chronic disease history as covariates (Table 5). Women who menopause less than one year had lower EQ5D index (β = -0.055, *p* = 0.050). Cigarette smoking and chronic diseases were associated with both EQ5D index (β = -0.135, *p* < 0.001, and β = -0.104, *p* < 0.001, respectively) and EQ5D-VAS (β = -0.057, *p* = 0.034, and β = -0.214, *p* < 0.001, respectively). Low education level and less physical activity were associated with impaired EQ5D index (β = -0.080, *p* = 0.003, and β = -0.056, *p* = 0.040, respectively).

**Table 5:** Multiple linear regression analysis using EQ5D index and EQ5D-VAS as dependent variables

	**Independent variable**	**B**	**SE B**	**β**	***P *** **value**
EQ5D index	Cigarette smoking	-0.033	0.007	-0.135	<0.001^***^
	Chronic diseases	-0.016	0.004	-0.104	<0.001^***^
	Education	-0.016	0.006	-0.080	0.003^**^
	Physical activity	0.002	0.001	0.056	0.040^*^
	Premenopausal	ref	-	-	-
	Menopause				
	0 ~ 1 years	-0.014	0.007	-0.055	0.050
	2 ~ 5 years	-0.007	0.005	-0.035	0.230
	>5 years	-0.009	0.005	-0.050	0.085
EQ5D-VAS scores	Chronic diseases	-7.475	0.931	-0.214	<0.001^***^
	Cigarette smoking	-3.127	1.470	-0.057	0.034^*^
	Premenopausal	ref	-	-	-
	Menopause				
	0 ~ 1 years	1.418	1.580	0.025	0.370
	2 ~ 5 years	1.736	1.204	0.041	0.150
	>5 years	-0.953	1.191	-0.023	0.424

*p < 0.05, **p < 0.01, ***p < 0.001.

### Health quality declined with time after menopause

**Figure 1:**
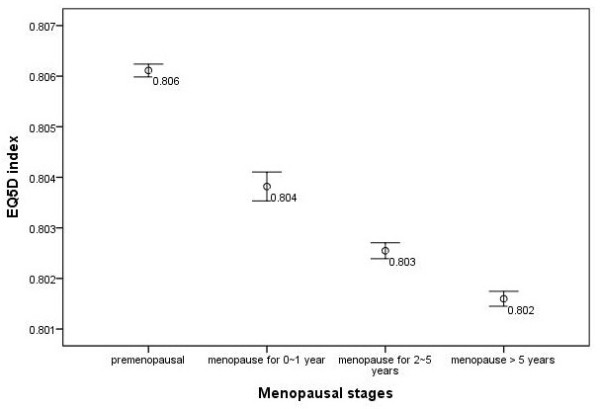
EQ5D index and its 95%CI in different menopause stages after adjusting for age.

**Figure 2:**
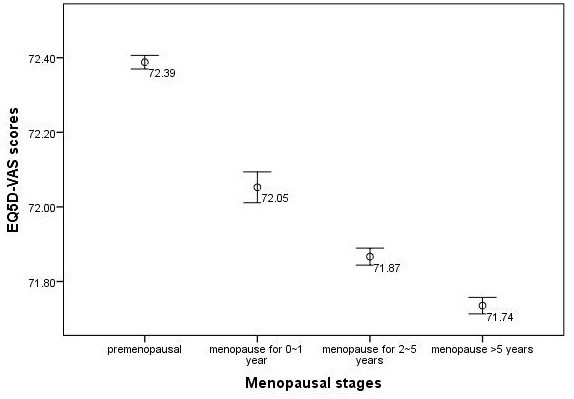
EQ5D-VAS scores and its 95%CI in different menopause stages after adjusting for age.

EQ5D index continued to decrease in each menopausal group categorized by menopausal duration after accounting for the effects of age. Premenopausal women has the highest EQ5D index (0.806) and EQ5D-VAS scores (72.39), women who had menopause less than 1 year has relatively lower EQ5D index (0.804) and EQ5D-VAS scores (72.05). EQ5D index and EQ5D-VAS scores continued to decrease in women who had menopause for 2 ~ 5 years (EQ5D index = 0.803, and EQ5D-VAS scores = 71.87) and both of the indicators reached the lowest values in the group of women who had menopause over 5 years (EQ5D index = 0.802, and EQ5D-VAS scores = 71.74) (Figures 1 and 2).

## Discussion

The Chinese women we evaluated had more mobility, usual activity and self-care problems after menopause, after accounting for the effects of general aging. Compared to premenopausal women, postmenopausal women had worse EQ5D index and EQ5D-VAS scores after adjusting for age. Low education level was associated with decreased mobility, usual activity problems, pain, and lower EQ5D index. Cigarette smoking and the presence of chronic diseases were risk factors for EQ5D index and EQ5D-VAS score. The strength of our study is that it is community-based one of few to address HRQOL middle-aged Chinese women. Since menopause symptoms may differ in China from other countries [12-14], this study provides useful information on the HRQOL related to menopause among Chinese women. Besides, stratified clustered sampling method and a relatively high response rate with the help of local government minimize sampling bias.

We found that women with stage +1b of menopause and had menopause for less than one year experienced more mobility problems and usual activity respectively than premenopausal women (Table 3). In addition, postmenopausal women had an impaired EQ5D index and EQ5D VAS scores compared to premenopausal women after adjusting for age (Table 4). These findings indicate that a reduction of physical function was mainly found within the first five years of menopause and menopause was related to HRQOL impairment. The results of our study are consistent with other researches. The SWAN study, a seven year longitudinal HRQOL study of 3302 middle-aged women who were experiencing menopausal transition, demonstrated significant reduction of physical function in late peri-menopausal and postmenopausal women [25]. The STRIDE longitudinal study [26] also demonstrated that women who were in late peri-menopausal (45.6, *p* ≤ 0.05) and early postmenopausal (45.4, *p* ≤ 0 .05) had lower physical health measured by RAND-36 compared to premenopausal women (47.1). Our results provide additional evidence of the negative impact of menopausal transition and early stage menopause on physical function.

However, a population-based survey based on 1,140 Greek middle-age women found no effect of menopause on the HRQOL [27]. Another 2-year follow up study on 734 premenopausal Taiwanese women also found no significant effect of menopausal transition on quality of life [19]. The discordance between the Taiwanese longitudinal study and our present may be attributed to different duration of menopause. In Taiwan, women experiencing menopause transitions were followed up for no more than 2 years. In our study, the mean elapsed time since menopause was 5.1 years, and women who had menopause for more than five years had the lowest HRQOL.

In the present study, we also found physical activity was associated with EQ5D index in our study. Several studies have also demonstrated the positive effect of physical activity on reducing menopause symptoms and improving HRQOL [28,29]. After 8-year follow up to 1,165 Finnish middle-aged women, JM Moilanen et al. demonstrated that women increase or remain stable physical activity had greater chances for improved HRQOL (OR = 1.49, 95%CI:1.23-1.80, *p* ≤ 0.001; OR = 1.46, 95%CI: 1.24-1.73, *p* ≤ 0.001, respectively) [30]. A randomized control trial (RCT) [31] evaluated the effect of physical exercise on HRQOL of postmenopausal women who had undergone hysterectomy, also demonstrated that physical exercises can reduce physical function and bodily pain symptoms due to menopause, and enhance HRQOL independent of hormone therapy. These evidences suggest that health promotion strategies on encouraging regular physical activities in menopausal women may improve their menopausal symptoms and HRQOL.

There were some limitations to our study. Firstly, as a cross-sectional study, it could not draw conclusions on the temporal changes in HRQOL in individual women, and causality can only be investigated to a limited extent. However, most of our results were consistent with other comparable longitude studies. Secondly, in our patients, it was required that menstruation was naturally stopped for at least one year without hormone therapy to be defined as postmenopausal. As cross-sectional study, it would be difficult to ascertain whether women who had menopause for less than a year were experiencing menopausal transition or in the early stage of postmenopausal. For this reason we could not consider women who had menopause for more than two menstrual cycles but less than one year as postmenopausal stage +1a. Thirdly, although the 3 level EQ-5D (EQ-5D-3 L) used to measure HRQOL in our survey has been widely used, the limited categories (i.e. levels) may restrict the reliable discriminant ability among different levels [32]. A 5 level EQ-5D (EQ-5D-5 L) is thought to have greater discrimination ability [33]. A Chinese version EQ-5D-5 L is a new and useful instrument for evaluating health status in Chinese people [34] and use of this measurement tool should be considered in future research. Finally, our analysis included only a few specific chronic disease conditions. It is possible that a more comprehensive look at chronic disease, such as osteoporosis, would better explain the effect of menopausal status on mobility and physical activity.

## Conclusion

Impaired EQ5D index, EQ5D-VAS scores and mobility problems were observed in postmenopausal women. Physical function reduction (mobility, usual activity, and self-care problems) was the main health problem in women within the first five years of menopause. Cigarette smoking, education level, physical activity and chronic disease were associated with HRQOL in middle-aged rural women.

## Competing interests

The authors declare that they have no competing interests.

## Authors’ contributions

KL, LH and YH conceived of the study, completed all statistical analyses, and drafted the manuscript; XT, NL, and JW participated in formulating the study, interpreting the data, and helped to draft the manuscript; JJL, LPY, HTX carried out the design of the study; JRL and YQW collected the data, and helped to revise the manuscript. RM contributed to the analysis and the interpretation of the study findings. All authors have read and approved the final manuscript.

## Pre-publication history

The pre-publication history for this paper can be accessed here:

http://www.biomedcentral.com/1472-6874/14/7/prepub
